# Case Report: First Two Identified Cases of Fabry Disease in Central Asia

**DOI:** 10.3389/fgene.2021.657824

**Published:** 2021-04-27

**Authors:** Francesca Cainelli, Dias Argandykov, Dauren Kaldarbekov, Murat Mukarov, Liên Tran Thi Phuong, Dominique P. Germain

**Affiliations:** ^1^Raffles Medical Group Clinic, Phnom Penh, Cambodia; ^2^Faculty of Medicine, University of Puthisastra, Phnom Penh, Cambodia; ^3^Private Practitioner, Nur-Sultan, Kazakhstan; ^4^School of Medicine, Nazarbayev University, Nur-Sultan, Kazakhstan; ^5^Department of Cardiology, National Research Cardiac Surgery Center, Nur-Sultan, Kazakhstan; ^6^French Referral Center for Fabry disease, Garches, France; ^7^Division of Medical Genetics, University of Versailles, Paris-Saclay University, Montigny, France

**Keywords:** Fabry disease, genotype-phenotype correlation, p.Arg49Gly variant, family screening, pedigree, Central Asia, Kazakhstan

## Abstract

**Background:** Fabry disease (FD, OMIM #301500) is a rare, progressive, X-linked inherited, genetic disease due to the functional deficiency of lysosomal α-galactosidase (α-GAL) that leads to the accumulation of glycosphingolipids (mainly globotriaosylceramide or Gb_3_) and its derivative globotriaosylsphingosine or lyso-Gb_3_. Classic FD is a multisystem disorder which initially presents in childhood with neuropathic pain and dermatological, gastrointestinal, ocular, and cochleo-vestibular manifestations. Over time, end-organ damage such as renal failure, cardiac arrhythmia and early stroke may develop leading to reduced life expectancy in the absence of specific treatment.

**Case presentation:** We describe two Kazakh patients who presented in adulthood with a delayed diagnosis. We conducted also a family screening through cascade genotyping.

**Conclusion:** This is the first description of cases of Fabry disease in Central Asia. An extensive family pedigree enabled the identification of ten additional family members. Patients with rare genetic diseases often experience substantial delays in diagnosis due to their rarity and non-specific symptoms, which can negatively impact their management and delay treatment. FD may be difficult to diagnose because of the non-specificity of its early and later-onset symptoms and its X-linked inheritance. Raising awareness of clinicians is important for earlier diagnosis and optimal outcome of specific therapies.

## Introduction

Fabry disease (FD, OMIM #301500) is a rare X-linked inherited genetic disease characterized by the functional deficiency of the lysosomal α-galactosidase (α-GAL) that leads to progressive, systemic accumulation of globotriaosylceramide (Gb_3_) and its derivative globotriaosylsphingosine (lyso-Gb_3_) in lysosomes of virtually all cell types (Brady et al., 1967). FD is caused by pathogenic variants in the *GLA* gene (HUGO Gene Nomenclature Committee ID: 4296; Gene Entrez: 2717; NCBI reference sequence: NM_000169.2) encoding α-galactosidase (α-GAL, Enzyme Commission number: EC 3.2.1.22; UniProt ID: P06280) resulting in its absent or substantially decreased activity (Germain et al., 2020). Glycolipids deposits occur in virtually all cells of the body among which vascular endothelial cells, renal endothelial and epithelial cells, cardiomyocytes and nerve cells. Classic Fabry disease exhibits a wide range of symptoms with onset during childhood for neuropathic pain (Politei et al., 2016), gastrointestinal disturbances (Auray-Blais et al., 2015), angiokeratoma, fatigue, impaired sweating, and heat/cold intolerance. Over time additional symptoms such as respiratory involvement (Magage et al., 2007) may develop while progression to end stage renal disease together with severe cardiac (Hagège et al., 2019) and cerebrovascular (Kolodny et al., 2015) complications contribute to reduced life expectancy (Desnick et al., 2003; Ortiz et al., 2018). Fabry disease can indeed be considered a multiorgan disease (Tuttolomondo et al., 2013). Female heterozygotes are also symptomatic especially when they exhibit skewed X chromosome inactivation profile with silencing of the wild-type allele (Wilcox et al., 2008; Echevarria et al., 2016). Beside the pathogenic variants leading to the classic phenotype of the disease, other *GLA* variants, while still pathogenic, have been found in association with residual α-Gal A activity and a usually milder, later-onset, predominantly cardiac phenotype of Fabry disease, including p.Phe113Leu, (Oliveira et al., 2019), p.Asn215Ser (Germain et al., 2018), p.Met296Ile, p.Gly328Arg, and the frequent IVS4+919G>A variant in Asia (Ishii et al., 2002; Hsu et al., 2016), while some *GLA* variants (e.g., p.Arg301Gln) may be more difficult to classify (Germain, 2001).

We describe the first two cases of Fabry disease in Central Asia (Kazakhstan); one was a hemizygous male and one a heterozygous female. After laboratory confirmation of the clinical suspicion, we conducted also an exhaustive family screening in the extended kindred of 115 individuals.

## Case Description

### Patient 1

**Figure 1:**
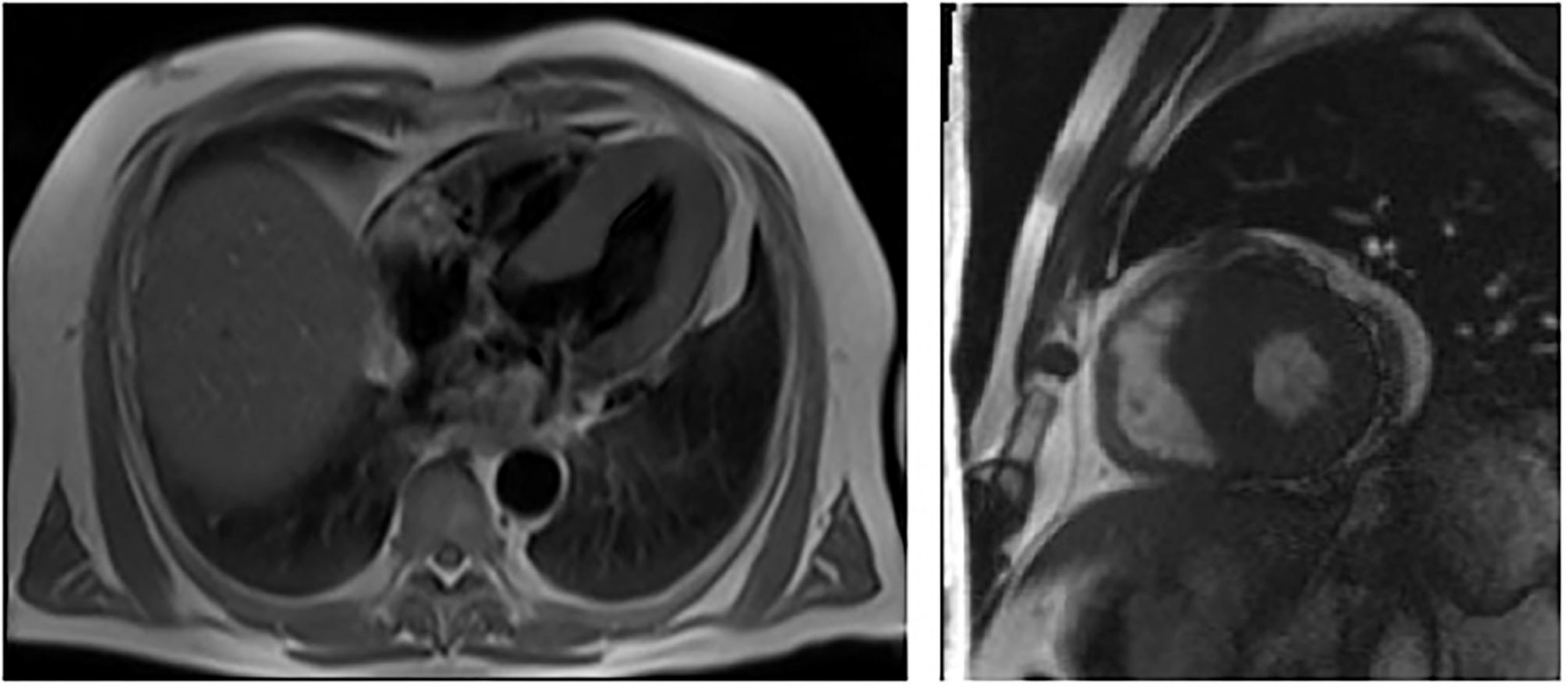
Cardiac MRI showing left ventricular hypertrophy with no late enhancement of Gadolinium in patient 1 (III – 13, proband).

Fifty-six-year-old Kazakh male patient, referred from South Kazakhstan to the Department of Cardiology of the National Research Cardiac Surgery Center in Nur-Sultan for repeated episodes of dizziness, malaise, dyspnea, and *angina pectoris*. The onset of symptoms (dizziness) occurred when the patient was 36 years of age. In 2012 he had been evaluated at the Cardiac Research Institute in Tomsk, Russia and had received a diagnosis of hypertrophic cardiomyopathy. One of the patient's brothers had died suddenly at the age of 38. Physical examination revealed an arterial blood pressure of 130/80 mm Hg, a heart rate of 56 bpm, normal oxygen saturation and body temperature and no dyspnea at rest; BMI was low (18.6 kg/m^2^). Small, dark reddish, raised skin lesions were clustered in the right flank area on the mid-axillary line. Routine laboratory tests showed mild anemia (Hb 12.7 g/dL) and 1+ proteinuria (33 mg/dL). Further blood tests revealed a markedly increased NT-ProBNP peptide (2,928 pg/ml; normal < 450 pg/ml) (Table 1). The electrocardiogram (ECG) showed sinus rhythm, PR interval of 120 ms, and signs of cardiac hypertrophy. An echocardiogram showed concentric hypertrophy of the left ventricle with near-normal systolic function. Cardiac magnetic resonance (CMR) imaging confirmed the concentric hypertrophic cardiomyopathy with end-diastolic thickness of the interventricular septum (IVS) and posterior wall (PW) of 23 and 13 mm, respectively. There was no late gadolinium enhancement (Figure 1). A carotid ultrasonography showed a narrowed portion of the internal carotid artery. An abdominal ultrasound revealed multiple small-sized cysts in both kidneys.

**Table 1:** Clinical and biochemical findings in the two patients.

**Patient characteristics**	**Patient 1**	**Patient 2**
Age	56	65
Sex	Male	Female
Presenting feature	Hypertrophic CM	Hypertrophic CM
Hb (g/dL)	12.7	14.1
Degree of Proteinuria (mg/dL)	33	No proteinuria
NT-ProBNP peptide (pg/ml; normal <450)	2,928	721
Interventricular septum (IVS) Thickness (mm)	23	19
Serum Creatinine (mg/dL)	1.10	0.63
Other findings	Angiokeratoma ICD	Multiple renal cysts

*CM, cardiomyopathy; NT-ProBNP, N-terminal pro b-type Natriuretic Peptide; ICD, Implantable Cardioverter Defibrillator*.

In the hypothesis of a familial form of sarcomeric hypertrophic cardiomyopathy without obstruction and due to the high risk of sudden cardiac death, a cardioverter-defibrillator was implanted. However, the concentric left ventricular hypertrophy and the characteristic cutaneous lesions prompted analysis of α-galactosidase activity and genetic testing for Fabry disease on dried blood spot (DBS). The analyses revealed a markedly diminished enzymatic activity (0.1 μmol/h/L; normal >1.2 μmol/h/L) and a missense pathogenetic variant, c.145C>G or p.(Arg49Gly) in *GLA* (Germain et al., 2002), thereby confirming the diagnosis of Fabry disease.

### Patient 2

**Figure 2:**
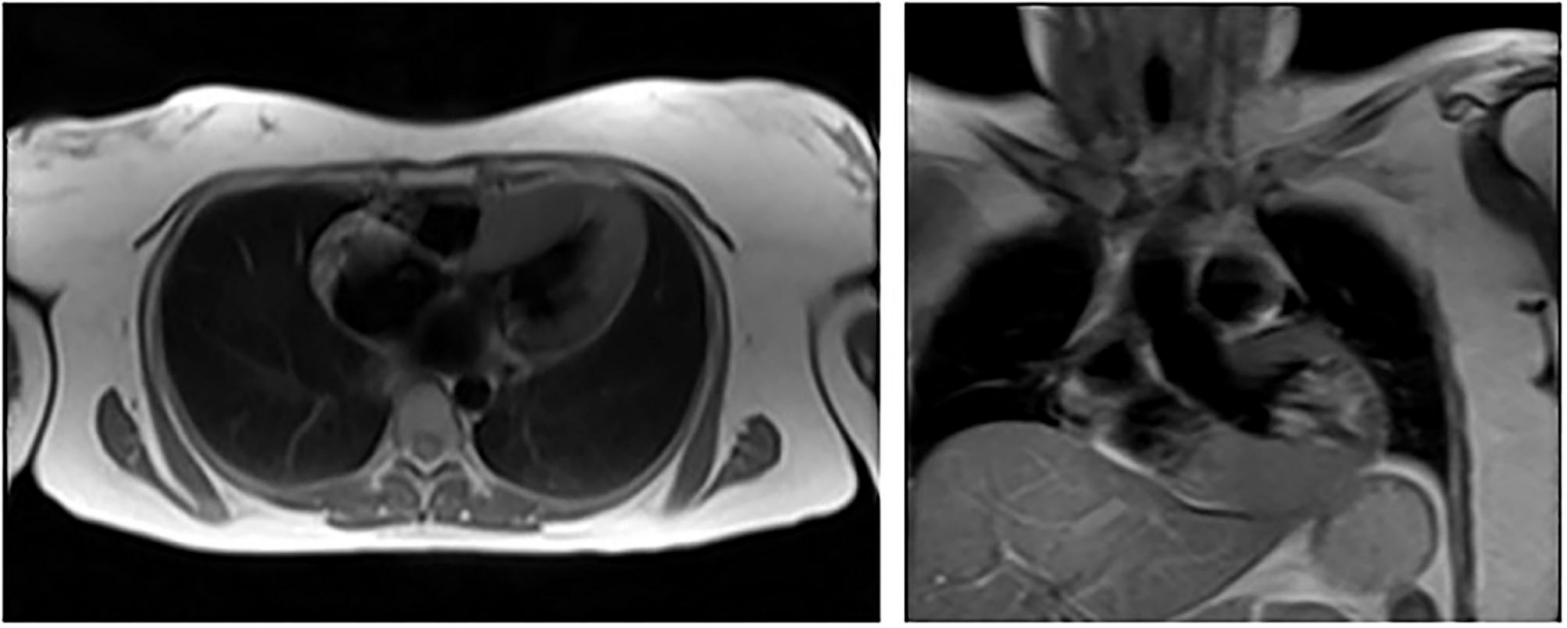
Cardiac MRI showing left ventricular hypertrophy in patient 2 (III – 16).

Proband's sister, 65-year-old Kazakh female, referred to the National Research Cardiac Surgery Center in February 2017 because of the possibility of an inherited form of hypertrophic cardiomyopathy. Since 2005 she had had dyspnea, retrosternal pain upon physical exertion and hypertension. ECG and echocardiography had shown left ventricular hypertrophy. She had also been evaluated at the Cardiac Research Institute in Tomsk in 2012, where a transesophageal echocardiography (TEE) had revealed unexplained IVS thickness (15 mm) but normal left ventricular posterior wall thickness (10 mm). Her blood pressure was normal (110/60 mmHg), her heart rate was 53 bpm, and an ECG was suggestive of left ventricular overload. An echocardiogram showed increased thickness (14 mm) of the interventricular septum without LV outflow tract obstruction. The LV ejection fraction was within the normal range. Laboratory tests revealed normal serum creatinine (0.63 mg/dL, normal <1.1), an increased level of NT-ProBNP peptide (721 pg/ml), elevated total cholesterol (6.45 mmol/L) and high LDL (4.99 mmol/L); there was no proteinuria (Table 1). A 24-h ambulatory ECG (Holter) monitoring did not show any episodes of tachyarrhythmia. CMR showed evidence of LV hypertrophy with IVS and PW thickness of 19 and 8 mm, respectively (Figure 2). Abdominal ultrasound showed multiple cysts in both kidneys. Cardiac catheterization did not identify any obstruction in the coronary arteries. Genetic and enzymatic analyses were performed and showed enzymatic activity of the α-galactosidase within normal values (1.6 μmols/h/L) but raised levels of globotriaosylsphingosine (lyso-Gb_3_) (5.8 ng/ml; 112 normal <3.5 ng/ml). Genetic analysis showed the same pathogenic variant in *GLA*: c.145C>G or p.(Arg49Gly).

### Pedigree Analysis

**Figure 3:**
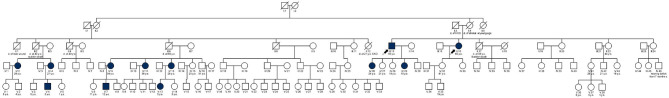
Family tree (pedigree). The index case and his sister are shown with arrows.

A pedigree of 115 individuals across four generations was drawn (Figure 3) with relevant clinical, enzymatic and genetic data (Table 2) (Germain et al., 2018). The family analysis allowed the identification of 31 women at risk of being heterozygotes for FD and 17 males at risk of being hemizygotes. Nineteen of them (15 female) have been tested so far and 10 (eight females) were found to be affected. The alpha-Galactosidase activity was low in eight family members (six females). The two males and the eight females bore the aforementioned pathogenic variant, p.Arg49Gly, a missense variation, previously associated with the classic phenotype of Fabry disease (Germain et al., 2002) and not amenable to migalastat (Benjamin et al., 2017). Although a female case (IV-13) with p.(Arg49Gly) had a normal alpha-Galactosidase activity on dried blood spot, her level of lyso-Gb_3_ was above the normal range (7.5 ng/mL). Likewise, another female case (V-13) with the *GLA* variant had a normal activity of the α-galactosidase but her plasma lyso-Gb_3_ was still above the normal range (5.5 ng/mL). In addition, among the affected individuals, one female (IV-9), aged 34, had end-stage renal disease (ESRD) and was on hemodialysis; one had severe joint pain and renal disease; one had tachyarrhythmia and two were asymptomatic. Importantly, the patient with renal failure had not received a diagnosis of FD before the current investigation; however, no kidney biopsy was performed, leaving unsolved the alternative hypothesis of a second nephropathy in addition to FD, responsible for ESRD. Molecular analysis of the *GLA* gene revealed the presence in all 10 affected relatives (eight females), of the p.Arg49Gly variant initially identified in the proband. Additional clinical investigations in members of the pedigree showed high mortality due to sudden death at a relatively young age in three males (III-2, III-4, and III-17) and one female (II-5) and 2 cases of stroke in one female (II-4) and one male (III-6), emphasizing the life-threatening complications associated with the classic phenotype of FD and the potential benefits that would have brought earlier diagnosis and pedigree analysis (Germain et al., 2018).

**Table 2:** GLA diagram of the family studied.

**Patient #**	**Gender& Age**	**GLA mutation**	**cDNA codon change**	**Enzyme activity (normal >1.2 μmol/L/h)**	**lyso-Gb3 (0–3.5 ng/mL)**	**Other symptoms**
III – 13	M/56	p.R49G	c.145C>G	0.1		Severe pain Cardiomyopathy Pacemaker Angiokeratoma
III – 16	F/65	p.R49G	c.145C>G	1.6	5.8	Cardiomyopathy Renal cysts
IV – 2	F/28	p.R49G	c.145C>G	0.7	4.5	WPW, Anemia
IV – 9	F/34	p.R49G	c.145C>G	0.3	13.4	CKD on HD Angiokeratoma
IV – 11	F/36	p.R49G	c.145C>G	0.4	10.2	Pain
IV – 13	F/28	p.R49G	c.145C>G	1.3	7.5	–
IV – 26	F/24	p.R49G	c.145C>G	0.8	5.3	Pain
IV – 29	F/10	p.R49G	c.145C>G	1.1	4.1	–
V – 6	F/11	–	–	2.1	2.2	–
V – 7	M/12	p.R49G	c.145C>G	0.2		Cerebral Palsy Pain
**Additional screening**						
III – 22	F/53	–	–	2.2	2	–
IV – 4	F/27	p.R49G	c.145C>G	0.7	10	Pain
IV – 41	F/25	–	–	3.6	1.6	–
IV – 42	M/20	–	–	2.1		–
IV – 43	F/18	–	–	2.9	1.6	–
V – 1	F/5	–	–	5.1	2	–
V – 2	M/4	–	–	3.6		–
V – 4	M/2	p.R49G	c.145C>G	0.3		–
V – 13	F/4	p.R49G	c.145C>G	2.3	5.5	–
V – 14	F/2	–	–	5	2.4	–
V – 36	F/4	–	–	2.4	1.4	–

## Discussion and Conclusion

The reported prevalence of FD is ~1:40,000 individuals worldwide, but newborn screening for FD has shown that the incidence is up to 1 in 3,300 or even 1 in 1,250 male births (Spada et al., 2006, Germain, 2010), the difference being possibly due to the existence of milder, later-onset variants of the disease (Germain et al., 2018; Oliveira et al., 2019) but also to the reports of *GLA* variants of uncertain clinical significance with increasing use of next-generation sequencing technologies including gene panels, exome and whole genome sequencing in newborns and high-risk patients with cardiomyopathy, early stroke or end-stage renal failure. We have described the first two cases of Fabry disease in Central Asia, where the prevalence of this disease is totally unknown.

A previously reported C-to-G transversion was detected at nucleotide c.145 in codon 49 of exon 1 (CGC → GGC), replacing a basic arginine with a neutral, polar glycine (p.Arg49Gly) in the index case, who was referred for cardiomyopathy. Fabry disease is inherited in an X-linked manner, therefore making males more susceptible to a severe phenotype (Hwu et al., 2009). Our patient #1 had overt cardiac manifestations of Fabry disease with minimal residual alpha-Gal A activity and left ventricular hypertrophy together with angiokeratomas.

Females heterozygotes for Fabry disease have long been considered asymptomatic carriers. However, a number of studies demonstrated much variability in clinical manifestations in heterozygous females, recently shown to be primarily due to variable X-chromosome inactivation profiles (Echevarria et al., 2016). Female patients identified through our family screening exhibited a significant phenotypic variability.

Family screening can greatly increase the number of patients diagnosed with rare genetic diseases and can facilitate earlier diagnosis which is particularly important for treatable genetic disorders, such as Fabry disease. Pedigree analysis in families with FD is valuable to identify, treat and monitor other possibly affected relatives. Hence, we constructed a pedigree for this first individual with Fabry disease identified in Kazakhstan. The construction and analysis of the pedigree of the kindred, consisting of 115 individuals, enabled the identification of at-risk heterozygous females and hemizygous males. The identification of affected individuals was done through biochemical analysis of the α-galactosidase enzyme and genetic testing. In male individuals, identification of the diminished level of the enzyme activity could be sufficient to make a diagnosis of Fabry disease, whereas in females molecular genetic testing remains mandatory to confirm the disease, as some heterozygous females might have a normal enzymatic activity as was the case for patient 2 (Deegan et al., 2006). Likewise, individuals IV-13 and V-13 were affected despite having normal enzymatic activity on DBS.

The etiology of the end-stage renal disease of the female case (IV-9) who had been on hemodialysis for a number of years, is not certain. It has been proposed that female patients with Fabry disease will progress to end-stage renal disease only if they exhibit a highly skewed X chromosome inactivation profile with predominant expression of the mutant *GLA* allele (Echevarria et al., 2016).

Enzyme replacement therapy (ERT) with recombinant human α-galactosidase has been available for the treatment of Fabry disease since 2001 in Europe and 2003 in the USA, and treatment outcomes depend on baseline patient characteristics and on the presence or absence of neutralizing antibodies (Wilcox et al., 2012; Germain et al., 2019a,b). ERT significantly decreases globotriaosylceramide levels in plasma, urine, and in different kidney, heart, and skin cell types, slows the decline in estimated glomerular filtration rate and reduces/stabilizes left ventricular mass and cardiac wall thickness. ERT also improves nervous system, gastrointestinal pain and quality of life outcomes (Ortiz et al., 2018; Germain et al., 2019a,b). Enzyme enhancement through pharmacological chaperon has been more recently approved for patients with Fabry disease and amenable *GLA* pathogenic variants (Germain et al., 2016; Benjamin et al., 2017). Amenability of the *GLA* variant to migalastat should always be carefully verified since discrepancies have been reported between patient outcomes and *in vitro* predictions (Lenders et al., 2019, 2020). The p.Arg49Gly variant is not amenable to migalastat and our patients have been initiated on enzyme replacement therapy with agalsidase beta at 1 mg/kg of body weight every other week.

Our report is the first description of cases of Fabry disease in Central Asia and demonstrates the importance of constructing a detailed family pedigree and conducting biochemical and genetic analyses in relatives of patients diagnosed with the disease. The screening of all family members is challenging; nevertheless, all efforts should be made to ensure that all at-risk groups of male and female relatives of an affected individual are timely screened to implement specific and symptomatic therapy before the disease progresses to its late stage.

### Patient Perspective

After receiving the diagnosis, the two patients felt relieved as they understood the cause of their illness and the possible cause of their brother's premature death, and they hope to get the right treatment for them and their relatives. They plan to write an application to the Regional Department of Health Care and get their costly therapy funded.

## Data Availability Statement

The raw data supporting the conclusions of this article will be made available by the authors, without undue reservation.

## Ethics Statement

Ethical review and approval was not required for the study on human participants in accordance with the local legislation and institutional requirements. The patients/participants provided their written informed consent to participate in this study. Written informed consent was obtained from the individual(s) for the publication of any potentially identifiable images or data included in this article.

## Author Contributions

FC designed the study and interpreted the data. DA wrote the first draft of the manuscript. DK and MM provided patients and relatives' data and contributed to the first draft. DG interpreted the data and wrote a revised version of the manuscript together with LT. All authors contributed to the article and approved the submitted version.

## Conflict of Interest

The authors declare that the research was conducted in the absence of any commercial or financial relationships that could be construed as a potential conflict of interest.
